# The Illusory Health Beliefs Scale: preliminary validation using exploratory factor and Rasch analysis

**DOI:** 10.3389/fpsyg.2024.1408734

**Published:** 2024-09-16

**Authors:** Andrew Denovan, Neil Dagnall, Kenneth Graham Drinkwater, Álex Escolà-Gascón

**Affiliations:** ^1^School of Psychology, Liverpool John Moores University, Liverpool, United Kingdom; ^2^Department of Psychology, Manchester Metropolitan University, Manchester, United Kingdom; ^3^Department of Quantitative Methods and Statistics, Comillas Pontifical University, Madrid, Spain

**Keywords:** convergent validity, illusory health beliefs, Illusory Health Beliefs Scale, questionnaire scrutiny, Rasch analysis

## Abstract

Illusory health beliefs are ill-founded, erroneous notions about well-being. They are important as they can influence allied attitudes, actions, and behaviors to the detriment of personal and societal welfare. Noting this, and the prevalence of paranormal beliefs in contemporary Western society, researchers developed the Paranormal Health Beliefs Scale (PHBS). Modification of the PHBS for use with a United Kingdom-based sample resulted in the instrument broadening to incorporate illusory rather than merely paranormal health beliefs. The present study psychometrically assessed the emergent Illusory Health Beliefs Scale (IHBS). The principal objective was to validate the IHBS using a large, representative sample. Eight hundred and fifty participants (360 males, 482 females, eight non-binary) completed the IHBS alongside instruments assessing theoretically associated constructs (i.e., magical thinking, faith in scientifically unsubstantiated notions, and forms of self-referential, intuitive causation). Exploratory factor analysis revealed the existence of six meaningful IHBS dimensions: Religious/Spiritual, Superstition, Precognitive, Health Myths, Skepticism, and Health Pseudoscience. The IHBS demonstrated satisfactory reliability and convergent validity with theoretically aligned constructs. Rasch analysis at the subscale level revealed good item/person fit and item/person reliability, unidimensionality, and equivalency of items across subgroups (gender and religious affiliation). Analysis confirmed the IHBS was an effective measure of illusory health beliefs. However, researchers should undertake further work to refine the scale and evaluate its performance across different samples and time points.

## Introduction

Paranormal belief endures within modern Western societies (see Dagnall et al., 2016, 2022b). Though advocacy varies as a function of survey and belief type, investigators consistently report prominent levels of supernatural credence within general samples. Across studies, Marks (2021) approximates endorsement is around 50% of the population appraised (Williams et al., 2022). Acknowledging this, and the potential of supernatural credence to influence well-being and lifestyle choices, Donizzetti and Petrillo (2017) created the Paranormal Health Beliefs Scale (PHBS). In the context of health, paranormal beliefs denote validation of notions that surpass the limits of what current scientific knowledge considers physically possible, and attribution of well-being to unknown powers/forces (e.g., ‘Guardian angels keep away illnesses’). Correspondingly, the PHBS is a self-report instrument that assesses individual propensity to substantiate supernatural-based views about wellbeing (illness origin, preventative health strategies, treatment, recovery, etc.). This perspective derived from the observation that ill-informed health-related actions and behaviors can prove detrimental to physical and mental welfare at individual and societal levels.

Although health-related paranormal beliefs are not typically associated with specific health-related outcomes they become maladaptive when they undermine or conflict with scientific and medical advice (Farias et al., 2013; Dagnall et al., 2019). This is especially true when dogmas embody false/flawed (illusory) ideations about welfare, which prevent engagement with established medical processes and procedures (Capone, 2016). For instance, Anderson and Emery (2014) observed that irrational health beliefs were a prognosticator of poorer adherence to rehabilitative care. Moreover, supernatural credence predicts positive attitudes to/and belief in complementary and alternative therapies and practices (Pettersen and Olsen, 2007; Van den Bulck and Custers, 2010). Complimentary treatment exists alongside established medicine, and alternative serve as replacements. Academics criticize complementary and alternative therapies and practices because they possess minimal empirical support and exist outside the recognized scientific paradigm (Li et al., 2018).

The operationalization of paranormal health beliefs as illusory and irrational concurs with the view that within general populations endorsement of supernatural phenomena represents a non-clinical manifestation of delusional thinking (Drinkwater et al., 2021; Irwin et al., 2012a,b). Specifically, errors in reality testing (Irwin, 2004), whereby believers base judgments on personal, subjective appraisal of data rather than objective evaluation of external evidence (Drinkwater et al., 2012). With reference to health beliefs, this manifests as the failure to adequately assess the legitimacy of self-generated hypotheses about health and wellbeing. This interpretation aligns with the supposition that paranormal health beliefs represent self-serving illusions (Donizzetti and Petrillo, 2017; Yarritu et al., 2015), which are personally efficacious (i.e., afford a sense of control and psychological reassurance) but medically ineffectual.

Although there is an absence of bespoke measures assessing paranormal health beliefs, researchers have included construct-related items within paranormal belief scales. For instance, Nixon’s Superstitions Scale (Nixon, 1925), the Supernaturalism Scale (Randall and Desrosiers, 1980), the Exeter Superstitions Questionnaire (Preece and Baxter, 2000), and the New Age Orientation Scale (Granqvist and Hagekull, 2001) contain statements referring to healing and disease. Relatedly, researchers in allied domains such as individual differences and psychopathology have acknowledged links between delusional thinking, magical ideation, and psychological adjustment. For example, the Schizotypal Questionnaire (Raine and Benishay, 1995), possesses a cognitive-perceptual factor, which evaluates odd beliefs and magical thinking, unusual perceptual experiences, ideas of reference, and paranoid ideation.

Regarding the PHBS, Petrillo and Donizzetti (2012) and Donizzetti and Petrillo (2017) created the instrument by producing a breadth of construct-related items, which they administered to 1,469 adolescents (Petrillo and Donizzetti, 2012). Exploratory and confirmatory factor analysis identified five belief types: religious (i.e., sacred notions of protection/recovery), superstitious (i.e., practices that guard individuals from health threats), Extraordinary Events (i.e., indefinite entities/events/forces that affect health), Parapsychological (i.e., mental energies that influence health), and Pseudo-scientific beliefs of a biomedical nature (i.e., threats to health arising from deviant or marginal social groups). Based on these outcomes, Petrillo and Donizzetti (2012) concluded that the multidimensional structure was psychometrically satisfactory.

In a follow-up study, Donizzetti and Petrillo (2017) validated the PHBS using a university-based sample. Confirmatory factor analysis (CFA) confirmed the original five-factor solution. Data best fitted an oblique five-factor model indicating that the PHBS comprised five empirically related dimensions. To substantiate dimension convergence, Donizzetti and Petrillo (2017) then performed a second-order CFA. Model fit was similar to the first-order model. The PHBS at both global and dimensional levels demonstrated concurrent validity via positive correlations with external Health Locus of Control Scale (HLCS) (Wallston et al., 1976) factors (i.e., God and Other). Donizzetti and Petrillo (2017) evidenced discriminant validity via negative correlations with the internal HLCS factor and non-significant relationships with General Self-Efficacy Scale (GSES) (Schwarzer and Jerusalem, 1995). These outcomes substantiated the initial study and demonstrated that the PHBS was appropriate for use with general populations.

Although the measurement of illusory health beliefs is conceptually and practically important, researchers have made only limited use of the PHBS (e.g., Rosa, 2018). From a measurement perspective, this is concerning because the instrument remains underdeveloped. Since the PHBS draws heavily on Italian culture, a particular issue is cultural specificity. Explicitly, items draw heavily on traditional religious (e.g., saints and holy relics) and societal (e.g., evil eye) icons/symbols. Noting this, Denovan et al. (2024), using cognitive interviewing, assessed item clarity and relevance for English-speaking participants. Cognitive interviewing accesses thought processes and perceptions by inviting respondents to verbalize thoughts as they advance through the scale. This facilitates identification of received meaning and item intelligibility.

Moreover, Denovan et al. (2024) used cognitive interviewing to assess the cultural applicability of the PHBS and identify scale improvements. This comprised interviews (*N* = 14) spread across two rounds. The first, which assessed comprehension, revealed issues with cultural references, wording, and phraseology. These problems undermined clarity and relevance, indicating the need for changes to item content and expression. The second round evaluated the effectiveness of modifications implemented following round one. Analysis found that although respondents still reported issues with ambiguity, alterations reduced terminology complexity.

Through PHBS revision, the researchers removed poorly performing statements. This in some instances left subscales with few items. Additionally, factors did not align well with extant academic literature (e.g., Pseudo-scientific, capturing health threats due to deviant/marginal groups). Accordingly, item enhancement drew on conceptually informed sources (e.g., traditional paranormal belief literature). This ensured that novel items (implemented prior to this study) effectively captured supernatural notions applied to health (e.g., psi), and ensured that items aligned with theoretical definitions. Additionally, correction of notable ambiguity concerns (e.g., the response option ‘Do not know’ to ‘Neither agree nor disagree’) occurred prior to the current study.

Overall, cognitive interviews advised that re-envisaging the scale as illusory (rather than paranormal) would increase measurement effectiveness of health beliefs. Furthermore, reconceptualization circumvented the need to determine whether subsumed scientifically unsubstantiated phenomena were paranormal or pseudoscientific in nature (see Dagnall et al., 2010a,b). This was an important advancement since the scale focuses on false health beliefs rather than supernatural credence *per se*.

### Current study

This paper further assessed the psychometric properties of the Illusory Health Beliefs Scale. Item refinement/development used the PHBS as a template, with additional items aligning with established aspects of paranormality (e.g., superstition, religiosity, precognition, psi) alongside unsupported convictions related to health, satisfying the definition of paranormal health beliefs as illusory with the potential to influence health practices/behavior (Petrillo and Donizzetti, 2012). Previous research (Denovan et al., 2024) informed the creation of a separate health pseudoscience subscale. In addition to traditional methods, Rasch analysis provided critical psychometric information about item performance (e.g., fit/appropriateness, difficulty), sample targeting, and dimensionality (see Duncan et al., 2003 for a review).

The focus of this research extends previous work. Specifically, Donizzetti and Petrillo (2017) demonstrated that the PHBS possessed satisfactory psychometric properties with an Italian sample. However, Donizzetti and Petrillo (2017) failed to implement tests of item difficulty and items were culturally specific. Moreover, the authors evidenced a relationship with important outcomes (e.g., illness) and emphasized that paranormal/illusory health beliefs could be useful for further appreciating factors that can influence health. Aside from Denovan et al. (2024), academics have not published work on this topic since. This is concerning because the culture-specific orientation of the scale hinders effective application within other contexts/cultures for assessing links between illusory health beliefs and related constructs. Accordingly, validation using empirically supported techniques (e.g., Rasch analysis) alongside construct/measure scrutiny in other contexts (an English-speaking context in this study) is critical for furthering research on the topic. Thus, development of a useful assessment tool for use with English-speaking samples will subsequently advance the research literature on illusory health belief. This includes exploration of latent structure, item/measure performance, and convergent validity using theoretically related constructs/measures (i.e., the PHBS, magical thinking, paranormal, pseudoscientific, and pro-scientific belief, and positive schizotypy).

It is not feasible to specify precise hypotheses concerning relationships with external criteria due to the absence of supporting literature. Using findings from the paranormal belief literature (e.g., Aarnio and Lindeman, 2005; Dagnall et al., 2022a; Irwin, 1990), illusory health beliefs should positively associate with all constructs apart from pro-scientific belief (should be negative). The authors anticipated strongest associations between the PHBS and the Illusory Health Beliefs Scale because the latter is a PHBS variant.

## Materials and methods

### Participants

The study sampled 850 UK-based respondents (360 males, 482 females, eight non-binary), mean age 41.29 (*SD* = 13.84), range 18–80. Demographic information comprised measures of educational level, self-defined ethnicity, religious affiliation, and degree of perceived religiosity, and spirituality (see Table 1). Recruitment occurred through Bilendi, an established supplier of quality data^1^. Participants were each allocated credits equalling £1.42 upon survey completion. The researchers instructed Bilendi to supply a representative sample of UK respondents aged 18 years and over from their participant panel. Panels provide data comparable to traditional approaches in quality (Kees et al., 2017). Data screening indicated satisfactory skewness and kurtosis between −2.0 to +2.0 among all study variables.

**Table 1:** Participant characteristics (*N* = 850).

Characteristic	(*n*)
Gender	
Male	360
Female	482
Non-binary	8
Educational level	
Postgraduate degree (inc. PGCE, Master’s, Doctorate, medical, or law degree)	157
Undergraduate/bachelor’s degree	258
College/further education	241
Secondary education	178
Other	16
Self-defined ethnicity	
White	691
Black	38
Asian	81
Mixed ethnic background	20
Prefer not to say	10
Other	10
Religious affiliation	
Catholic/Roman Catholic	144
Buddhist	10
Hindu	21
Jehovah’s Witness	5
Jewish	4
Methodist	20
Muslim	55
Orthodox	17
Protestant	109
No religion	402
Other	63
Perceived religiosity	
Very religious	64
Moderately religious	157
Slightly religious	192
Not at all religious	437
Perceived spirituality	
Very spiritual	99
Moderately spiritual	182
Slightly spiritual	244
Not at all spiritual	325

### Measures

This study used a range of psychometrically validated self-report measures.

#### Illusory Health Beliefs Scale (IHBS)

The IHBS (Denovan et al., 2024) is a 41-item scale that appraises illusory health convictions. This is a considerably revised variant of the PHBS (as detailed in the Introduction). Higher scores indicate greater endorsement of scientifically unsubstantiated notions (e.g., ‘Curses may cause illness’). The IHBS includes a 10-item subscale, which evaluates pseudoscientific beliefs relating to health (e.g., ‘Osteopathy encourages the body to heal itself by manipulating specific muscle tissue and bones’). Formation of this subscale occurred due to recommendations from cognitive interviewing (cf. Denovan et al., 2024). This ‘Health Pseudoscience’ subscale adapted items from previous measures (Fasce and Picό, 2019; Torres et al., 2020), focusing specifically on the interplay of pseudoscience and health. IHBS items appear as statements alongside a five-point Likert response format (1 = *Strongly Disagree* to 5 = *Strongly Agree*).

#### Convergent validity

To assess convergent validity, participants completed the Paranormal Health Beliefs Scale (Petrillo and Donizzetti, 2012), the Illusory Beliefs Inventory (Kingdon et al., 2012), the Revised Paranormal Belief Scale (Tobacyk, 2004), the Pseudoscientific Belief Scale (Fasce et al., 2021), the Belief in Science Scale (Farias et al., 2013), and the Unusual Experiences subscale of the short Oxford-Liverpool Inventory of Feelings and Experiences (Mason et al., 2005).

#### Paranormal Health Beliefs Scale (PHBS)

The PHBS is a 31-item scale, assessing the inclination to endorse supernatural notions concerning health. Higher scores depict greater belief in anomalous health practices/behaviors. Items use a statement-based format (e.g., ‘Cases of healing due to strength of faith do exist’) and participants respond using a five-point Likert scale (1 = *Strongly Disagree* to 5 = *Strongly Agree*). The measure comprises five subscales (Religious Beliefs, Superstitious Beliefs, Extraordinary Events Beliefs, Parapsychological Beliefs, and Pseudo-scientific Beliefs). Satisfactory reliability exists (Donizzetti and Petrillo, 2017). The current study excluded the Pseudo-scientific Beliefs subscale due to concerns with item content for a UK-based sample (see Denovan et al., 2024).

#### Illusory Beliefs Inventory (IBI)

The IBI consists of 24 items measuring beliefs associated with magical thinking. The measure contains three subfactors. Explicitly, Magical Beliefs, which refer to general belief in magic, Spirituality relating to general religious philosophy, beliefs in a spiritual presence and defiance of scientific explanations, and Thought-Action Fusion, which is the convention that an inseparable link exists between thought and action. Higher scores overall reflect greater endorsement of events occurring due to magical occurrences, belief in a higher power/guiding spirit, and belief in the strength of thoughts/dreams/intuitions predicting occurrences. Within the IBI items appear as statements (e.g., ‘If I think too much about something it will happen’). Participants respond using a 5-point scale (1 *= Strongly Disagree*, 5 *= Strongly Agree*). Kingdon et al. (2012) reported excellent alpha reliability with a non-clinical sample.

#### Revised Paranormal Belief Scale (RPBS)

The RBPS is a 26-item measure, which assesses validation of paranormal/supernatural phenomena with a 7-point Likert scale (1 = *Strongly Disagree*, 7 = *Strongly Agree*). Higher scores indicate greater anomalous belief. Included are seven subscales (Traditional Religious Belief, Psi, Witchcraft, Superstition, Spiritualism, Extraordinary Lifeforms, and Precognition). This study used the total RPBS score. The RPBS typically exhibits excellent internal consistency (e.g., Drinkwater et al., 2017).

#### Revised Pseudoscientific Belief Scale (Pseudo-R)

The Pseudo-R uses 19 items to assess inclination to endorse pseudoscientific notions [e.g., ‘Neuro-linguistic programming (NLP) is accepted as part of psychology’]. Greater scores infer stronger convictions regarding practices/beliefs that claim to be scientific (but lack scientific validation). Participants record responses on a 5-point Likert scale (1 = *Strongly Disagree* to 5 = *Strongly Agree*). Pseudo-R possesses excellent internal consistency (Fasce et al., 2021).

#### Belief in Science Scale (BIS)

The BIS examines the extent to which individuals regard science as a superior source of knowledge, using 10 items (e.g., ‘The scientific method is the only reliable path to knowledge’) alongside a 6-point Likert scale (1 = *Strongly Disagree* to 6 = *Strongly Agree*). Greater scores reflect a tendency to view science as a robust form of information provision. The BIS possesses excellent internal reliability (Dagnall et al., 2019).

#### Unusual Experiences Subscale (UnExp)

The UnExp is a subscale from the short Oxford-Liverpool Inventory of Feelings and Experiences, which assesses positive schizotypy (hallucinations, magical thinking) with 12 items (e.g., ‘Are your thoughts sometimes so strong that you can almost hear them?’), and a ‘Yes/No’ response format. Higher scores indicate a greater presence of positive schizotypy symptoms. Satisfactory reliability exists (Mason et al., 2005).

Within the present study scales were internally reliable: PHBS (Religious Beliefs, *α* = 0.95, *ω* = 0.95; Superstitious Beliefs, *α* = 0.93, *ω* = 0.93; Extraordinary Events Beliefs, *α* = 0.90, *ω* = 0.90; Parapsychological Beliefs, *α* = 0.91, *ω* = 0.91); IBI (Magical Beliefs, *α* = 0.86, *ω* = 0.86; Spirituality, *α* = 0.81, *ω* = 0.78; Thought-Action Fusion, *α* = 0.86, *ω* = 0.86); RPBS (*α* = 0.96, *ω* = 0.96); Pseudo-R, *α* = 0.88, *ω* = 0.92; BIS, *α* = 0.93, *ω* = 0.93; and UnExp, *α* = 0.85, *ω* = 0.85.

### Procedure

Before taking part, participants read the information sheet detailing the study background alongside study procedures. All participants provided informed consent before completing the study. This included ticking/clicking a box within the online survey confirming that they understood the study’s purpose and agreed to participation. Instruction informed that they could withdraw from the study at any point during completion. Additional instructions emphasized to participants to answer truthfully, take their time, and complete all questions. A forced response option and an inbuilt randomizer minimized incomplete responses and order effects of questionnaires in the online survey. Prior to completing study measures, participants provided demographic information. The Manchester Metropolitan University Ethics Committee (EthOS ID #52313) provided ethical approval.

### Data analysis plan

Validation of the IHBS progressed through iterative stages. Firstly, exploratory factor analysis (EFA) with Principal Axis Factoring and oblique rotation, explored underlying structure using three criteria: Velicer’s minimum average partial (MAP) test, scree plot, and an eigenvalue ≥1. Velicer’s MAP test determines the number of underlying factors by computing partial correlations among residuals until no further common variance remains (O’Connor, 2000). EFA focused initially on IHBS items, and secondly on the ‘Health Pseudoscience’ subscale. This was necessary because although pseudoscience endorsement is related to paranormal belief, the constructs differ. For instance, they are differentially related to ontological confusion (i.e., conflation of mental and physical phenomena) (Lobato et al., 2014). While ontological confusion predicts paranormal belief, it does not predict pseudoscience endorsement. Conceptual differences explain why there is only a medium association (*r* = 0.36) between paranormal and pseudoscientific belief.

Recognizing differences, the authors presented Health Pseudoscience alongside IHBS items. This enabled parallel assessment of constructs without potential theoretical obtrusion. Moreover, concurrent assessment was advisable from a psychometric perspective since EFA performed on Health Pseudoscience and IHBS items would potentially confound factor identification/coherence. The fact that EFA is exploratory and atheoretical accentuates this concern (Tabachnick and Fidell, 2001). Hence, though related and commonly bracketed as epistemically unwarranted beliefs (Lobato et al., 2014), the constructs required separating for EFA.

Rasch analysis (Winsteps) subsequently examined rating scale efficacy, reliability, dimensionality, item fit and difficulty, and differential item functioning (DIF) (Denovan et al., 2022). Rasch evaluation (Rasch, 1960) was necessary because critics regard classical test theory (CTT) as limited. Explicitly, they disagree with the assumptions that test scores, in the absence of error, are accurate, and measurement error is random. Modern test theory instead contends that error occurs systematically as a function of individual ability and item difficulty. Acknowledging this, Rasch modeling calculates expected item responses. At the polytomous level (Rasch rating scale model) it applies to data with two or more ordinal categories (e.g., Likert type), as in this study. The polytomous model is advantageous because it offers estimates of person locations, item difficulties, and thresholds (fixed across items). This information designates item efficacy (i.e., discriminatory power).

Effectiveness of the rating scale was determined via monotonic increases in response category usage alongside Infit and Outfit (required to be between 0.5 and 2.0; Wright and Linacre, 1994). Item separation/reliability and person separation/reliability indicated reliability. A threshold of 0.7 exists for reliability (Fisher, 1992), and separation indicates the degree of participant or item spread on the ability continuum in addition to the extent of distinct levels of item/person ability (Bond and Fox, 2015). Values >1.5 suggest that items/samples separate into at least two levels (e.g., low, and high ‘complexity/ability’).

Principal Components Analysis of the residuals (PCAR) examined unidimensionality (a key assumption of Rasch analysis) via the following criteria: ≥40% of variance accounted for by the Rasch dimension; ≤15% of variance accounted for by the first contrast in the residuals; and a first contrast eigenvalue <3 (Areepattamannil and Khine, 2018). Additional dimensions exist if these criteria are not satisfied. Infit and Outfit Mean square error (MNSQ) determined whether items ‘misfit’ in relation to the measure. A misfitting item indicates that this is tapping into something distinct from the remainder items on the scale.

Examination of item ‘difficulty’ vs. person ‘ability’ used person-item maps. Efficacious measures should be able to differentiate participants along the ability continuum, with ideal targeting represented by the item mean corresponding with the person mean. DIF assessed equivalency of items across subgroups (gender and religious affiliation). A DIF contrast >0.64 alongside a significant Mantel–Haenszel *p*-value indicates that subgroups vary in their interpretation of items (Linacre, 2015). Finally, internal reliability testing occurred prior to examining convergent validity associations (using Pearson’s *r*) with theoretically related measures.

## Results

### Exploratory factor analysis

The MAP test recommended extraction of four (Revised MAP Test) and six (Original MAP Test) factors, whereas EFA suggested five factors. Explicitly, the eigenvalue criterion and scree plot (Supplementary material 1). Comparison of competing solutions indicated the five-factor solution yielded the most homogeneous and interpretable factors with minimal cross-loadings (i.e., only item 30, ‘Dreams about the future can suggest ways to avoid illness’, loaded above 0.4 on multiple factors). In comparison, the four-factor and six-factor solutions revealed cross-loadings on three different items. Moreover, the four-factor model excluded a factor with a meaningful eigenvalue, and the six-factor model produced a factor with an unsatisfactory eigenvalue. Accordingly, the researchers selected the five-factor solution, which persisted after removal of item 30. The model accounted for 63.20% of variance and demonstrated satisfactory sampling adequacy, Kaiser–Meyer–Olkin (KMO) of 0.97, and a suitable item correlation matrix (Bartlett’s Test of Sphericity *p* < 0.001). One item loaded below 0.4 (0.39), retained due to its proximity to the threshold.

Labels derived from conceptual interpretation of factor content. Factor 1, ‘Religious/Spiritual’ (9 items), captured holy/spiritual beliefs about health (eigenvalue = 18.05, 44.19% variance). Factor 2, ‘Superstition’ (12 items), comprised health-related items linked to prediction and ritual (eigenvalue = 2.31, 4.54% variance). The third factor, ‘Precognitive’ (6 items), contained items referencing the ability to influence/affect health via psychic forces (eigenvalue = 2.11, 4.14% variance), and Factor 4, ‘Health Myths’, (5 items), consisted of well-being falsehoods (eigenvalue = 1.49, 2.69% variance). Lastly, Factor 5, ‘Skepticism’, (5 items), included negatively worded items that reflected disbelief in illusory health beliefs (eigenvalue of 1.32, 2.0% variance) (Table 2).

**Table 2:** Psychometric properties of the Illusory Health Beliefs Scale at the item level.

Item	Factor	EFA loading	Infit MNSQ	Outfit MNSQ	Difficulty	DIF contrast gender	DIF contrast religion	PTMEA Corr.
1. Illness can be overcome by psychic forces	Precognitive	0.46	0.96	0.99	0.36	−0.29	0.00	0.72
2. It is better to avoid medical appointments (for example, visiting the doctor or dentist) on certain dates, such as Friday 13th	Superstition	0.70	1.37	1.49	0.72	−0.26	0.10	0.65
3. People can influence health through psychic forces	Precognitive	0.54	0.91	0.90	0.33	−0.34	0.06	0.74
4. The soul or spirit can influence health	Precognitive	0.62	1.14	1.14	−0.51	0.09	0.00	0.75
5. Horoscopes provide important health-related information	Superstition	0.81	0.93	0.90	0.23	−0.24	−0.04	0.76
6. Religious faith heals diseases	Relig./Spir.	0.88	0.81	0.77	0.26	−0.15	−0.18	0.81
7. Superstitions, such as saying ‘touch wood’ or actually touching wood, ward off threats to health	Superstition	0.74	1.02	1.10	−0.01	0.20	0.30	0.77
8. Holy water protects against illness and disease	Relig./Spir.	0.78	0.83	0.73	0.55	−0.12	−0.02	0.79
9. Cases of healing due to strength of religious faith exist	Relig./Spir.	0.87	0.87	0.87	−0.38	−0.03	−0.13	0.84
10. Curses may cause illness	Relig./Spir.	0.50	1.21	1.24	0.16	0.00	0.29	0.77
11. States of illness can facilitate the separation of the spirit from the body	Precognitive	0.46	0.87	0.85	0.25	0.03	−0.29	0.75
12. During acute health conditions, it is possible to feel that one’s own spirit is floating out of one’s own body or to perceive one’s own body from an external position	Precognitive	0.80	1.11	1.12	−0.11	0.09	0.24	0.73
13. Superstitions associated with bad luck, such as breaking a mirror, have no impact on health (R)	Skepticism	0.50	1.14	1.10	−0.17	0.00	0.19	0.67
14. Health is in the hands of God	Relig./Spir.	0.86	1.08	1.04	−0.10	0.00	−0.59	0.81
15. Wearing an amulet or a lucky charm helps to keep one healthy	Superstition	0.57	1.0	1.07	−0.19	−0.13	−0.23	0.79
16. Psychic forces can provoke changes in health conditions (such as an increase in body temperature or a quickening of the heartbeat)	Precognitive	0.59	0.80	0.79	0.30	−0.02	0.00	0.76
17. The powers of the mind cannot cure people of illness (R)	Skepticism	0.69	0.87	0.88	−0.34	−0.11	0.18	0.70
18. Only science and modern medicine can explain why people contract illness (R)	Skepticism	0.58	0.94	0.98	−0.01	0.11	−0.24	0.65
19. Some psychics can accurately predict illness	Precognitive	0.61	0.92	0.99	0.09	0.22	0.07	0.75
20. I believe that ‘eating an apple a day will keep the doctor away’	Health Myth	0.39	1.05	1.10	0.34	−0.18	−0.02	0.65
21. It is important to ‘feed a cold and starve a fever’	Health Myth	0.49	0.97	1.01	−0.24	0.02	0.04	0.64
22. People have visions about things that can affect their health	Precognitive	0.62	1.03	1.02	−0.43	−0.11	0.21	0.75
23. A person’s future health has nothing to do with their zodiac sign (R)	Skepticism	0.47	1.12	1.03	0.52	0.00	−0.18	0.64
24. The power of prayer can cure disease	Relig./Spir.	0.92	0.84	0.80	−0.26	0.18	−0.37	0.84
25. Guardian angels or other spiritual forces can protect against illness	Relig./Spir.	0.44	1.05	1.10	−0.21	0.41	0.37	0.81
26. Fortune telling (using a crystal ball, reading tea leaves) can predict future health	Superstition	0.67	0.82	0.82	−0.18	0.14	0.00	0.81
27. Hunches and intuitions about illness are not just coincidences	Precognitive	0.72	1.40	1.49	−0.59	0.25	0.39	0.68
28. Horoscopes accurately predict your health	Superstition	0.82	0.76	0.68	0.31	−0.23	−0.11	0.78
29. ‘Cracking your knuckles’ causes arthritis	Health Myth	0.44	1.05	1.03	0.29	−0.08	0.00	0.65
30. *Dreams about the future suggest ways to avoid illness*								
31. Sitting too close to the television will harm your eyesight	Health Myth	0.59	1.17	1.14	−0.67	0.12	0.27	0.67
32. If you ‘catch a chill, you will catch a cold’	Health Myth	0.59	0.84	0.83	0.15	0.21	−0.11	0.71
33. Religious faith contributes to a person’s general health	Relig./Spir.	0.61	1.37	1.56	−0.31	−0.16	0.36	0.77
34. It is possible to have visions about becoming ill which come true	Precognitive	0.52	1.05	1.05	−0.12	0.00	0.09	0.73
35. Some people have a special gift to heal other people from illness simply by touching them	Precognitive	0.45	0.99	0.91	0.32	0.16	−0.44	0.73
36. Health conditions can be treated with spiritual healing	Precognitive	0.48	0.81	0.78	0.09	−0.06	−0.33	0.77
37. Breaking glass or a mirror does not bode well for health	Superstition	0.61	1.38	1.48	−0.22	0.03	0.15	0.75
38. Card reading (tarot cards) can tell a lot about a person and their future health	Superstition	0.65	0.79	0.78	−0.27	0.35	0.10	0.83
39. Healing prayer from a spiritual healer can cure disease	Relig./Spir.	0.53	0.93	0.97	0.29	−0.10	0.23	0.79
40. Radiation absorbed from using a mobile phone can cause cancer	Health Myth	0.40	0.90	0.91	0.12	−0.10	−0.15	0.68
41. Some people can predict your future health by looking at the lines on your palm	Superstition	0.54	0.94	0.95	−0.39	0.05	−0.24	0.81
Item in italics removed following EFA; (R) denotes reverse-keyed item; Relig./Spir., Religious/Spiritual.								

Item	Factor	EFA loading	Infit MNSQ	Outfit MNSQ	Difficulty	DIF contrast gender	DIF contrast religion	PTMEA Corr.
Health Pseudoscience subscale								
1. Possessing a positive and optimistic attitude helps to prevent cancer	Health Pseudoscience	0.51	1.35	1.45	0.93	−0.19	−0.39	0.58
2. Osteopathy encourages the body to heal itself by manipulating specific muscle tissue and bones	Health Pseudoscience	0.68	0.77	0.82	0.21	−0.26	0.00	0.69
3. Physical illnesses can be cured through the manipulation and channeling of forces and energies (Reiki)	Health Pseudoscience	0.64	0.92	0.89	1.02	−0.08	−0.14	0.67
4. Homeopathic remedies, where individuals are given diluted substances to trigger natural healing mechanisms, effectively complement the treatment of diseases	Health Pseudoscience	0.68	0.87	0.89	0.52	0.16	−0.22	0.69
5. Pain problems can be successfully treated by inserting needles in specific parts of the body (acupuncture)	Health Pseudoscience	0.73	0.86	0.85	−0.23	0.15	0.00	0.71
6. Nutritional supplements, such as vitamins or minerals, enhance health and prevent diseases	Health Pseudoscience	0.54	1.21	1.19	−0.62	0.06	0.35	0.59
7. Toxic substances can be effectively eliminated from the body through detox therapies and/or diets	Health Pseudoscience	0.71	0.84	0.87	−0.01	−0.22	−0.06	0.70
8. Stomach ulcers can be caused by stress	Health Pseudoscience	0.54	1.30	1.24	−1.01	0.15	0.31	0.60
9. Chiropractic (manipulation of the joints) is a scientifically supported form of physiotherapy	Health Pseudoscience	0.63	1.04	1.06	−0.40	0.00	0.23	0.66
10. Applying pressure to specific parts of the feet (Reflexology) can relieve pain in connected areas of the body, such as organs, muscles, and joints	Health Pseudoscience	0.72	0.85	0.84	−0.41	0.37	0.15	0.71

(R) denotes reverse-keyed item.

The MAP Test designated a one-factor solution for the pseudoscience subscale. Satisfactory KMO (0.90) and item correlations (Bartlett’s Sphericity *p* < 0.001) existed. Factor loadings were above 0.4 (i.e., 0.51 and greater), and the solution explained 47% of data variance. The authors subsequently labeled the factor ‘Health Pseudoscience.’

Factors partially aligned with the original PHBS structure, specifically Religious/Spiritual, Superstition, and Precognitive. Additional factors referenced misconceptions/myths about health and doubt regarding the veracity of anomalous practices. This latter dimension of Skepticism was unanticipated. However, this provides a useful counterpoint to antiscientific convictions regarding health. The Health Pseudoscience subscale items also coalesced as one subscale, as expected.

Factors from the IHBS (excluding Skepticism) and Health Pseudoscience were highly positively correlated. Religious/Spiritual associations: Superstition, 0.72; Precognitive, 0.82; Health Myths, 0.61; Health Pseudoscience, 0.50. Superstition associations: Precognitive, 0.78; Health Myths, 0.63; Health Pseudoscience, 0.43. Precognitive associations: Health Myths, 0.65, and Health Pseudoscience, 0.63. Health Myths: Health Pseudoscience, 0.59.

Skepticism demonstrated an inconsistent pattern of weak correlations. Specifically, negative associations with Religious/Spiritual, Superstition, and Precognitive, and positive associations with Health Myths and Health Pseudoscience.

### Rasch analysis

Existence of large correlations among IHBS factors and Health Pseudoscience potentially point to the presence of a global factor underpinning the questionnaire. Prior to computing Rasch analysis separately for IHBS factors and Health Pseudoscience, a PCAR investigated the existence of subdimensions for the total questionnaire. This provided independent confirmation of the dimensionality indicated by EFA (Franchignoni et al., 2013). An eigenvalue of the first contrast >3.0 infers that there is another dimension in the measurement instrument (Linacre, 2012). Findings revealed a first contrast eigenvalue of 8.0, supporting multidimensionality.

Applying Rasch analysis to IHBS factors and Health Pseudoscience revealed that the rating scale functioned appropriately, evidenced by monotonic increases in average measures from response category 1 (*Strongly Disagree*) to 5 (*Strongly Agree*), alongside Infit and Outfit MNSQ results between 0.5 and 2.0 (Supplementary material 2).

Measurement reliability was good across IHBS factors and Health Pseudoscience (Religious/Spiritual = 0.97, item separation = 6.12; Superstition = 0.98, item separation = 6.36; Precognitive = 0.98, item separation = 7.36; Health Myths = 0.99, item separation = 8.58; Skepticism = 0.99, item separation = 8.56; Health Pseudoscience = 1.0, item separation = 14.14). Person reliability was also satisfactory for all scales apart from Skepticism (Religious/Spiritual = 0.81, Superstition = 0.74, Precognitive = 0.85, Health Myths = 0.74, Skepticism = 0.58, Health Pseudoscience = 0.84). Moreover, a person separation >1.5 existed for all measures aside from Skepticism (1.18), indicating separation of participants into more than one ability level (Linacre, 2012). The results for Skepticism potentially suggested the need for more items.

The PCAR supported unidimensionality for most scales (Religious/Spiritual %age explained = 60%, eigenvalue = 13.5, %age of variance explained by first contrast = 6.8%, eigenvalue = 1.5; Superstition %age explained = 54%, eigenvalue = 10.6, %age of variance explained by first contrast = 9.4%, eigenvalue = 1.8; Precognitive %age explained = 54.7%, eigenvalue = 14.5, %age of variance explained by first contrast = 7.3%, eigenvalue = 1.9; Health Myths %age explained = 43.9%, eigenvalue = 4.7, %age of variance explained by first contrast = 14%, eigenvalue = 1.5; Health Pseudoscience %age explained = 50.8%, eigenvalue = 10.3, %age of variance explained by first contrast = 10.2%, eigenvalue = 2.1). Skepticism, however, revealed unacceptable explained variance in the first contrast, but acceptable results in relation to the other criteria for unidimensionality (Skepticism %age explained = 40%, eigenvalue = 2.6, %age of variance explained by first contrast = 24.5%, eigenvalue = 1.6).

**Figure 1:**
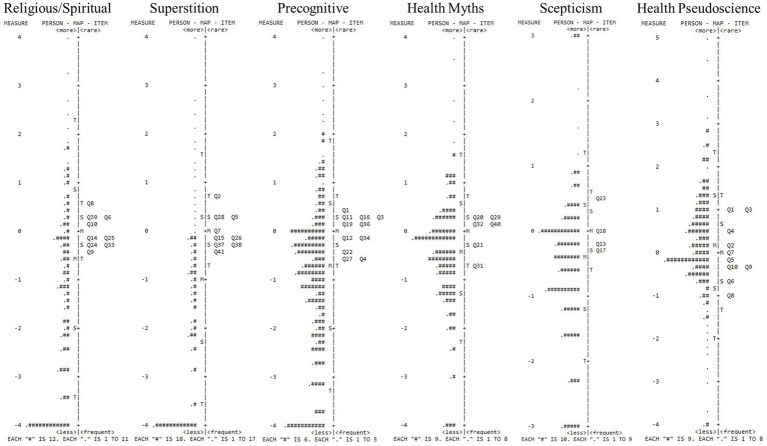
Person-item maps of the IHBS. Participants are on the left of the dashed lines (more able are located at the top of the map). Items are on the right of the dashed lines (more difficult items are located at the top of the map. M = Mean person ability of mean item difficulty; S = one standard deviation; T = two standard deviations.

Items across scales demonstrated satisfactory Infit and Outfit MNSQ (between 0.5 and 2.0) alongside positive and strong Point Measure Correlations (>0.40), inferring a lack of unsuitable or misfitting items (Table 2). Figure 1 shows person ability and item difficulty. For Religious/Spiritual, Superstition, and Precognitive, mean endorsement was lower than average item difficulty, with a noticeable cluster of participants exhibiting low Rasch scaled scores. This indicated that respondents were unlikely to endorse these scales. Health Myths, Skepticism, and Health Pseudoscience evidenced a more even spread of participants relative to items. However, there still existed only a few participants with high scores. Item difficulty calibration indicated no discernible differences (i.e., denoted by a similar spread of items around the mean).

DIF analyses relative to gender (male vs. female) and religion (religious affiliation vs. no religious affiliation) displayed no meaningful concerns. Specifically, although significant Mantel–Haenszel values existed, DIF contrasts <0.64 logits (Table 2) inferred subgroups attributed similar meaning to the items.

### Reliability and validity

Following the guidelines of Taber (2018), alpha and omega reliability for Religious/Spiritual (*α* = 0.95, *ω* = 0.95), Superstition (*α* = 0.94, *ω* = 0.94), and Precognitive (*α* = 0.94, *ω* = 0.94) were excellent. Health Myths (*α* = 0.78, *ω* = 0.78) and Health Pseudoscience (*α* = 0.87, *ω* = 0.87) demonstrated good reliability. Skepticism evidenced moderate reliability (*α* = 0.63, *ω* = 0.62). Convergent validity analysis revealed significant moderate to large associations concerning IHBS factors and Health Pseudoscience with all variables aside from Belief in Science. Indeed, this demonstrated a similar pattern of associations with IHBS factors as Skepticism (Table 3).

**Table 3:** Correlations between IHBS factors and the variables used to establish convergent validity.

Variables	Relig	Super	Precog	Myths	Scept	Pseudo
PHBS						
Religious beliefs	0.73**	0.69**	0.83**	0.58**	−0.09**	0.61**
Superstitious beliefs	0.87**	0.76**	0.77**	0.58**	−0.10**	0.47**
Extraordinary events beliefs	0.71**	0.86**	0.69**	0.57**	−0.11**	0.36**
Parapsychological beliefs	0.71**	0.78**	0.76**	0.59**	−0.07*	0.49**
IBI						
Magical beliefs	0.67**	0.80**	0.74**	0.55**	−0.14**	0.46**
Spirituality	0.66**	0.40**	0.59**	0.33**	−0.27**	0.33**
Thought-action fusion	0.63**	0.64**	0.72**	0.56**	−0.01	0.51**
Total	0.77**	0.73**	0.80**	0.56**	−0.18**	0.50**
RPBS						
Paranormal Belief	0.77**	0.78**	0.82**	0.60**	−0.08*	0.54**
Pseudo-R						
Pseudoscience	0.64**	0.63**	0.75**	0.60**	−0.01	0.68**
BIS						
Belief in science	−0.21**	−0.01	−0.15**	0.06	0.30**	0.09*
UnExp						
Unusual experiences	0.42**	0.50**	0.51**	0.36**	0.02	0.34**

Relig, religious/spiritual; Super, superstition; Precog, precognitive; Myths, health myths; Scept, skepticism; Pseudo, health pseudoscience; *p < 0.05, **p < 0.001.

As predicted, IHBS paranormal-based factors (Religious/Spiritual, Superstition, Precognitive) correlated the strongest with PHBS factors. Health Pseudoscience correlated the strongest with Pseudo-R, which was appropriate. Some inconsistencies occurred, however, such as Pseudo-R demonstrating the strongest association with Precognitive. This is potentially due to an emphasis on ontological confusion with some of the Pseudo-R items (e.g., ‘The collective memory inherited and shared by the organisms belonging to the same species (‘morphic field’ or also ‘morphic resonance’) explains several biological phenomena’), in addition to an item directly referencing precognition [i.e., ‘It has been scientifically proven that some people have extrasensory abilities (such as telepathy or precognition)’].

## Discussion

Analysis identified five conceptually coherent, internally consistent factors within the IHBS (Denovan et al., 2024). Four highly positively correlated factors, Religious/Spiritual, Superstition, Precognitive, and Health Myths, represented the propensity to endorse distinct but related forms of illusory health belief. The fifth factor Skepticism denoted the tendency to reject unsubstantiated notions about wellbeing. Skepticism demonstrated inconsistent relationships with other IHBS factors. The factor was weakly negatively associated with Religious/Spiritual, Superstition, and Precognitive, and weakly positively associated with Health Myths. Additionally, Skepticism was weakly positively correlated with Health Pseudoscience. These relationships were explicable via factor content. Religious/Spiritual, Superstition, and Precognitive assessed ideations allied to traditional paranormal beliefs (Drinkwater et al., 2018), whereas Health Myths evaluates misapplication of scientific ideas and principles (Dagnall et al., 2019; Farias et al., 2013). The fact that factors strongly positively correlated with Health Pseudoscience demonstrated the importance of this construct (i.e., the tendency to erroneously regard theories, assumptions, and methods as scientific) within the IBHS (see Boudry et al., 2015).

Convergent validity confirmed the pattern of intra IHBS and Health Pseudoscience relationships. Explicitly, Religious/Spiritual, Superstition, Precognitive, and Health Myths (i.e., factors allied to endorsement of illusory beliefs), and Health Pseudoscience correlated positively with scores on IBI subscales (Magical Beliefs, Spirituality, and Thought-Action and Fusion), RPBS, PHBS subscales (Religious, Superstitious, Extraordinary Events, and parapsychological), Pseudo-R, and UnExp. These relationships demonstrated that the IHBS and Health Pseudoscience possessed content validity. Specifically, that they shared variance with scales, which independently assess belief in the paranormal, magical thinking, faith in scientifically unsubstantiated notions, and forms of self-referential, intuitive causation. Collectively, these constructs are crucial to the formation and maintenance of illusory health beliefs (Boudry et al., 2015). Skepticism was positively related to BIS.

Regarding discriminant validity, IHBS subscales assessing endorsement of illusory beliefs were either weakly negatively (i.e., Religious/Spiritual and Precognitive) or not correlated (Superstition and Health Myths) with BIS. Health Pseudoscience was weakly positively correlated with BIS. Finally, Skepticism was either negatively associated with (IBI, RPBS, and PHBS subscales) or failed to correlate (Pseudo-R, and UnExp) with concurrent measures assessing inclination to magical thinking and endorsing scientifically unsubstantiated forms of causation.

Rasch analysis confirmed the psychometric functioning of the IHBS factors and Health Pseudoscience subscale. Moreover, support for more than one dimension existed. Though, initial inter-factor associations indicated some degree of commonality. It would be important for future research to determine the source of this. For instance, testing the presence of a higher-order structure vs. meaningful shared variance using latent modeling techniques. Given that these possess shared qualities of antiscientific (and epistemically unwarranted) beliefs, the authors anticipate a relationship between factors. In comparison with Health Pseudoscience, IHBS beliefs index stronger associations. This is consistent with the observation that IHBS beliefs are more paranormal in nature and share features such as ontological confusion (Lobato et al., 2014).

Contrasting with the IHBS factors and Health Pseudoscience, Skepticism demonstrated poorer psychometric performance. This factor was unexpected during EFA, but potentially provides a meaningful counterpoint to antiscientific convictions. Indeed, the concept of skepticism is central to scientific and antiscientific belief (cf. French, 2015). However, at present the findings infer the need to develop this factor further, by refining and developing novel items.

IHBS endorsement was low. This reflected the polarizing nature of item content, which captures ‘believers’ vs. ‘non-believers’. This is not a concern *per se*, given general population samples typically reflect 50% or lower endorsement of illusory/supernatural/religious beliefs (Williams et al., 2022; World Values Survey, 2023). Rather, a measure capturing these beliefs within a general population sample should report polarity in endorsement, providing preliminary evidence of suitable item-sample targeting.

### Limitations

Despite establishing validity and internal reliability further investigations should test the stability of the measures in independent samples and across time. Furthermore, the present study was cross-sectional meaning that the investigators collected data at one point in time. While the researchers employed procedural remedies to prevent common method variance (i.e., randomized scale presentation order and instructed participants that scales assessed distinct constructs, see Drinkwater et al., 2020, 2024) it is still necessary to assess scale robustness and verify external reliability (i.e., temporal stability). Consequently, ensuing psychometric evaluation should appraise test–retest reliability. This will ensure that scores are consistent and replicated across time. This is important since reliability across multiple trials, settings, and respondents is often absent or poorly reported (Dagnall et al., 2023). Moreover, now convergent validity has been established relative to mental health/paranormal-based scales, in additon to verifying latent structure (e.g., via confirmatory factor analysis), a further necessary step is to examine IHBS associations with health-based measures.

The present IHBS and Health Pseudoscience subscale combined are lengthy, this potentially restricts their inclusion in large test batteries. Acknowledging this, ensuing studies should create short abridged versions. This process will reduce cognitive load and increase accessibility. However, scale refinement is an iterative process that requires assessment of item performance across a range of samples. This is also necessary to ensure that the evolving brief measures adequately assess construct breadth.

## Data Availability

The raw data supporting the conclusions of this article will be made available by the authors, without undue reservation.

## Footnotes

^1^ https://www.bilendi.co.uk/
