# Supplementary figures and images for: The Illusory Health Beliefs Scale: preliminary validation using exploratory factor and Rasch analysis
Source: Front Psychol. 2024 Sep 16;15:1408734. doi: 10.3389/fpsyg.2024.1408734 (PMC11440939; doi:10.3389/fpsyg.2024.1408734)

**Appendix S1** Scree Plot of the IHBS


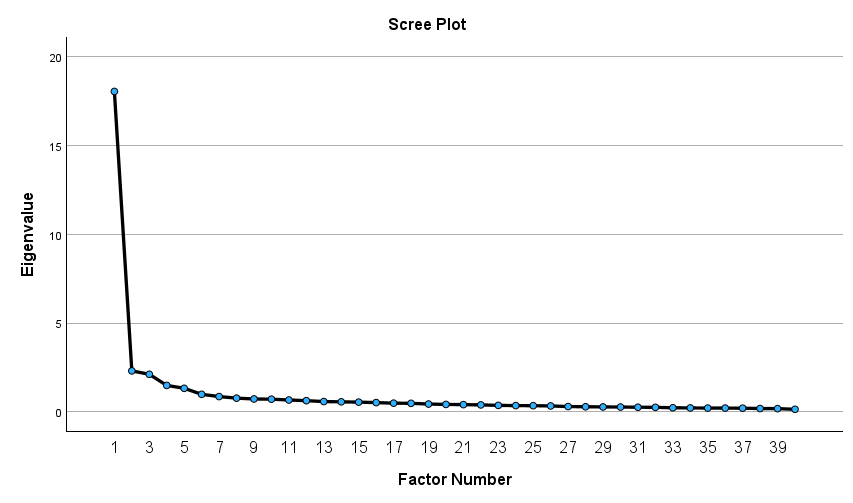

Supplement: Supplementary file 1 [file Table_1.docx]
