# Supplementary material for: The Illusory Health Beliefs Scale: preliminary validation using exploratory factor and Rasch analysis
Source: Front Psychol. 2024 Sep 16;15:1408734. doi: 10.3389/fpsyg.2024.1408734 (PMC11440939; doi:10.3389/fpsyg.2024.1408734)
Supplement: Supplementary file 2 [file Table_2.docx]

**Appendix S2** Rating scale effectiveness of the IHBS

| IHBS Scale | Category | Total count (%age) | Infit MNSQ | Outfit MNSQ |
| --- | --- | --- | --- | --- |
| Religious/Spiritual |  |  |  |  |
|  | 1 Strongly disagree | 3054 (40) | 1.0 | 1.05 |
|  | 2 Disagree | 1061 (14) | .97 | .92 |
|  | 3 Neither agree nor disgaree | 1804 (24) | .84 | .85 |
|  | 4 Agree | 1118 (15) | 1.16 | 1.19 |
|  | 5 Strongly agree | 541 (7) | 1.06 | 1.10 |
| Superstition |  |  |  |  |
|  | 1 Strongly disagree | 3834 (51) | .92 | .96 |
|  | 2 Disagree | 1154 (15) | .88 | .90 |
|  | 3 Neither agree nor disgaree | 1537 (20) | .79 | .81 |
|  | 4 Agree | 755 (10) | 1.10 | 1.23 |
|  | 5 Strongly agree | 298 (4) | 1.50 | 1.60 |
| Precognitive |  |  |  |  |
|  | 1 Strongly disagree | 3311 (33) | .91 | .98 |
|  | 2 Disagree | 1508 (15) | .99 | .91 |
|  | 3 Neither agree nor disgaree | 2843 (28) | .82 | .80 |
|  | 4 Agree | 1885 (19) | 1.11 | 1.14 |
|  | 5 Strongly agree | 557 (6) | 1.32 | 1.30 |

**Supplementary Table 1** *continued*

| IHBS Scale | Category | Total count (%age) | Infit MNSQ | Outfit MNSQ |
| --- | --- | --- | --- | --- |
| Health Myths |  |  |  |  |
|  | 1 Strongly disagree | 1139 (23) | .98 | 1.01 |
|  | 2 Disagree | 858 (17) | 1.04 | 1.08 |
|  | 3 Neither agree nor disgaree | 1632 (32) | .89 | .87 |
|  | 4 Agree | 1055 (21) | 1.04 | 1.05 |
|  | 5 Strongly agree | 368 (7) | 1.02 | 1.01 |
| Scepticism |  |  |  |  |
|  | 1 Strongly disagree | 1027 (30) | 1.06 | 1.06 |
|  | 2 Disagree | 770 (23) | .83 | .91 |
|  | 3 Neither agree nor disgaree | 740 (22) | .80 | .71 |
|  | 4 Agree | 402 (12) | .96 | .92 |
|  | 5 Strongly agree | 429 (13) | 1.16 | 1.21 |
| Health Pseudoscience |  |  |  |  |
|  | 1 Strongly disagree | 1105 (13) | 1.01 | 1.09 |
|  | 2 Disagree | 871 (10) | 1.08 | 1.12 |
|  | 3 Neither agree nor disgaree | 2591 (31) | .89 | .89 |
|  | 4 Agree | 2675 (32) | .92 | .92 |
|  | 5 Strongly agree | 1178 (14) | 1.08 | 1.04 |
